# Pathways to care of patients with mental health problems in Bangladesh

**DOI:** 10.1186/s13033-018-0218-y

**Published:** 2018-07-18

**Authors:** Nazmun Nahar Nuri, Malabika Sarker, Helal Uddin Ahmed, Mohammad Didar Hossain, Claudia Beiersmann, Albrecht Jahn

**Affiliations:** 1 Institute of Public Health, Ruprecht-Karls-Universität Heidelberg, INF 130/3, 69120 Heidelberg, Germany; 2 James P. Grant School of Public Health, BRAC University, 66 Mohakhali, Dhaka, 1212 Bangladesh; 3 National Institute of Mental Health, Sher-e-Bangla Nagar, Dhaka, 1200 Bangladesh; 4 Foundation for Advancement of Innovations in Technology and Health, 1/15A Iqbal Road, Dhaka, 1207 Bangladesh

**Keywords:** Pathway, Pathways to mental health care, Mental illness, Psychiatric disorders, Mental health, Bangladesh

## Abstract

**Background:**

Health systems in Bangladesh are not fully organized to provide optimal care services to patients with mental health problems. There is both a lack of resources and a disproportional distribution of the available resources. To design an equitable health system and plan interventions to improve access to care, a better understanding of mental health care-seeking behavior and care pathways are crucial.

**Methods:**

A facility-based cross-sectional study was conducted using a mixed-method design at the National Institute of Mental Health (NIMH), in Bangladesh. A total of 40 patients (or their attendants) visiting the outpatient department of NIMH were selected by purposive sampling.

**Results:**

As their first contact point for care services, 27.5% of the patients consulted a psychiatric care provider, 30% went to non-medical provider, and the majority, 42.5%, went to non-psychiatric medical care providers. Only 32.5% of the patients had been advised to go to NIMH by a private physician, hospital personnel or psychiatrist. Among all individual categories of providers, private psychiatrists were the most frequent caregivers (n = 12), followed by traditional healers (n = 9). A total of 70% of the patients had chosen a provider within 20 km. In three out of four of the cases, the family had decided on the first provider. From the start of the symptoms the median delay in the first contact with any provider was 6 months, and in reaching any psychiatric care provider was 1 year. The most common reasons for a delay in seeking care were a lack of knowledge about mental health problems, a lack of information about the place for appropriate care, and not considering the problem as serious enough to seek care. Each of those reasons were mentioned by one in every four respondents.

**Conclusions:**

The majority of the patients with mental health problems in Bangladesh access various categories of providers before reaching a psychiatric care provider, and use a diverse range of pathways and loops, which results in a delay or missing appropriate care. We hope that our findings are useful for planning interventions to improve access to mental health care in general, in Bangladesh, and improving referral policies and structures in particular.

## Background

Mental ill-health represents a large part of the disease burden of non-communicable diseases [1]. According to the World Health Organization (WHO) more than 450 million people worldwide are suffering from mental, neurological, or psychosocial disorders, and this number is constantly growing [1]. In Europe, neuropsychiatric disorders rank as the leading cause of years lived with disability, and account for 36.1% of all causes [2]. Of ten leading causes of disability, mental disorders represented four. A total of 12% of the global burden of disease is due to mental and behavioral disorders [1], and more than 70% of this burden lies in low- and middle-income countries [3].

In spite of the immense suffering from mental health problems, a huge gap between treatment need and provision exists across the world as a consequence of an inadequate response to the real burden of mental illness [4]. Two-thirds of all persons suffering from mental illness worldwide go untreated, and in low-resource countries, this figure is estimated to be higher than 90% [5]. Compared to low- and medium-income countries, high-income countries have 20 times more beds in community-based inpatient units, 30 times more admissions, a 40 times higher rate of patients care in outpatient facilities, 66 times more community outpatient contacts and 15 times more mental health staff at the outpatient level [6]. A better understanding of mental health care-seeking behavior and the care pathway is crucial for designing an equitable health system and planning interventions to improve access.

In Bangladesh, 16.1% of adults [7] and 15.2% of 5–10 year old children [8] are suffering from some type of mental health problem. The estimated population of Bangladesh was 161 million in 2015 [9], so it comprises a huge disease burden if the prevalence is converted to the total number of people affected in the country. WHO estimates that neuropsychiatric disorders in Bangladesh contribute to 11.2% of the total disease burden [10]. Still, like many other low-income countries, mental health has not received adequate attention from the Bangladeshi government. Bangladesh’s national health budget was USD 2.3 billion [11], and only 0.44% was allocated for mental health [10]. Bangladesh spends USD 16.20 per person per year on health and 64% comes from patient out-of-pocket payments. No social health insurance scheme in the country covers mental health care [11].

Bangladesh has one mental health institute, named National Institute of Mental Health (NIMH) [11], which has a 200 bed specialized mental hospital attached to it [12]. In addition to the NIMH, there is another 500-bed mental hospital (Pabna Mental Hospital, located 162 km west of Dhaka), 31 community-based psychiatric inpatient units, 15 beds in forensic inpatients units, 3900 beds in residential facilities (e.g., homes for the destitute, inpatient detoxification centers and homes for people with mental disability) and 50 outpatient mental health facilities in the country. Most of those mental health facilities are clustered in urban areas, particularly in metropolitan cities. The density of psychiatric beds in or around the capital Dhaka is five times higher than that in the entire country [13], even though 70% of Bangladeshi population lives in rural areas and the country comprises an area of 147,570 km^2^ [11].

Counting all psychiatric beds available in community psychiatric units and mental hospitals, Bangladesh has only one psychiatric bed for every 100,000 persons [13] and this is significantly lower than a median of 72/100,000 in the European countries [2]. There are 0.073 psychiatrists/100,000 population in Bangladesh [13] compared to 12.9/100,000 in EU countries [2]. The critical shortage of trained health care providers in Bangladesh is resulting in a widespread increase in informal providers (largely untrained providers of Western, homeopathic and traditional medicine) as alternative sources of care. Although not regulated by government authorities, these are the principal providers for poor Bangladeshi populations, especially in remote rural and hard-to-reach areas [11]. Moreover, a huge stigma and misconception regarding mental illness exist in Bangladeshi society. As a consequence, access to mental health care is inequitable and insufficient [11].

Unlike EU countries [2], there appears to be hardly any substantial mental health care available at primary or secondary health facilities in Bangladesh. Primary health care personnel lack the skills to detect and treat mental health patients, and referrals of patients with mental health problems to psychiatric providers by general practitioners or other health care providers is almost non-existent [14]. Hence the choice of provider depends on various factors including the explanatory model held by patients and their families, as well as the availability of care providers [14].

This study aims to understand the care pathways of patients with mental illness in Bangladesh, particularly considering the provider consulted, referral pattern, factors influencing the choice of provider, delays in care seeking and the reasons behind the delays. We believe that this vital information would enable health service planners to take appropriate action to reduce the gap between the need and the receipt of psychiatric care across the country.

## Methods

### Study design and study site

This study was a facility-based cross sectional study using mixed methods and conducted at NIMH, the only mental health institute of Bangladesh having academic functionalities. NIMH is situated in the capital city and caters to the whole country. Since its establishment in 2001, this hospital has provided care to 286,215 patients in OPD, 21,785 patients in the inpatient department and 16,420 patients in the emergency department. In 2015 alone, 42,703 patients received services in the NIMH OPD and 2501 patients in the inpatient department [12].

### Study sample

This study was conducted between July and September 2016. A total of 40 patients or their attendants visiting the NIMH OPD were interviewed. Four respondents were selected in each of the ten ICD 10 (10th revision of the WHO International Classification of Diseases) categories for mental disorders by purposive sampling. The diagnoses of patients taking part in our study was made by the OPD providers. Where possible, we recruited two males and two female patients as well as two adult and two minor patients in each of the ICD 10 categories. Since this was not possible for all ICD 10 categories, we ultimately recruited 23 male and 17 female patients including and 23 adult and 17 minor patients.

Mental stability of the patients selected for our study was determined by the OPD provider in charge. If a patient’s mental state was not sufficiently stable to allow participation in the interview, we interviewed his/her attendant. In total, 13 patients and 27 attendants (close family member, e.g., spouse, parent, sibling) were interviewed. An adult attendant was interviewed for all minors under 18 years of age. The inclusion criteria were: a patient/his or her attendant who had had a consultation with a doctor at the NIMH OPD at least once, and who agreed to participate in this study.

It was assumed that data saturation would be achieved at the end of 40 interviews. Various types of mental health problems cause distinct levels of disability and require specific care. So we decided to include a similar number of respondents from each of the ICD 10 categories for mental disorders to ensure diversity and representativeness.

### Study tool

We used a semi-structured in-depth interview guide and a structured questionnaire to collect data. Both were developed in English and then translated into Bengali by the first author, who is a native Bengali speaker. One native Bangladeshi mental health expert and two Bangladeshi public health experts reviewed the translated tools. The in-depth interviews focused on the onset of symptoms, respondents’ perceptions regarding the illness, influences on their choice of provider, personal experiences and constraints at each step of care seeking, and the reasons for delays in care seeking. The structured questionnaire was developed in line with concepts of other pathway-to-care studies [15–17] following the methodology of a WHO pathway study and its pathway encounter form. Our questionnaire focused on information on the patients’ demographic backgrounds, every provider consulted for the current mental health problem, primary reasons for care seeking, distance to each provider, source of information/referral to each provider, duration and type of treatment/advice received from each provider, impact of treatment on the symptoms at each step, and time passed without treatment before each step of care seeking.

### Data analysis

Quantitative data was collected by the questionnaire in Bengali and was entered directly into an English format by the first author. Descriptive statistics were evaluated using Stata version 14. The in-depth interviews conducted in Bengali were transcribed directly into English by the first author. Qualitative data was analyzed using the qualitative data analysis software NVivo 11. Based on a content analysis approach [18–21], a combination of deductive and inductive coding was performed. Deductive codes were based on the questionnaire and interview guide. The inductive codes were generated from the information derived from the interviews. In this way common themes and divergent views were able to be identified. The codes were cross checked by two of the co-authors and were modified where necessary. From the codes, the themes were developed and included referral patterns, parallel consultations from various providers, switching to a non-medical care provider after consulting a psychiatrist, reasons behind choosing a provider, and reasons behind delays in seeking care.

## Results

A combination of quantitative and qualitative data was obtained and our findings have been presented below.

### Demographic characteristics of the patients

Table 1 presents patients’ demographic characteristics. Of a total of 40 patients, 23 (57.5%) were adults. The mean age of respondents was 25.5 years (range 3–65 years). Slightly more than half (57.5%) of all patients were male, and 52.5% lived in the Dhaka urban area. The majority of the adult patients (69.6%) were unemployed. Slightly more than half of the adult patients (56.5%) were married, living with a spouse, and had between a 6th and 12th grade of schooling. Just over half (58.8%) of the minor patients were attending school.

**Table 1:** Patient demographic characteristics

Variable	N	Percentage (%)
Age		
< 18 years	17	42.5
18 years or above	23	57.5
Sex		
Male	23	57.5
Female	17	42.5
Marital status of adult patients (N = 23)		
Single	5	22.2
Residing with the spouse	13	56.5
Abandoned/divorced/widowed	5	22.2
Place of residence		
Urban	21	52.5
Rural	11	27.5
Semi-urban	8	20
Employment status of adult patients (N = 23)		
Currently working	7	30.4
Not working	16	69.6
Educational status		
Among adults (N = 23)		
Up to 5th grade	7	30.4
6th–12th grade	13	56.5
> 12th grade	3	13
Among minors (N = 17)		
< 5 years of age	3	17.6
Attending school	10	58.8
Dropped out (after 5th grade)	1	5.9
Never attended school	3	17.6

### The pathway of care seeking

#### First step

**Fig. 1:**
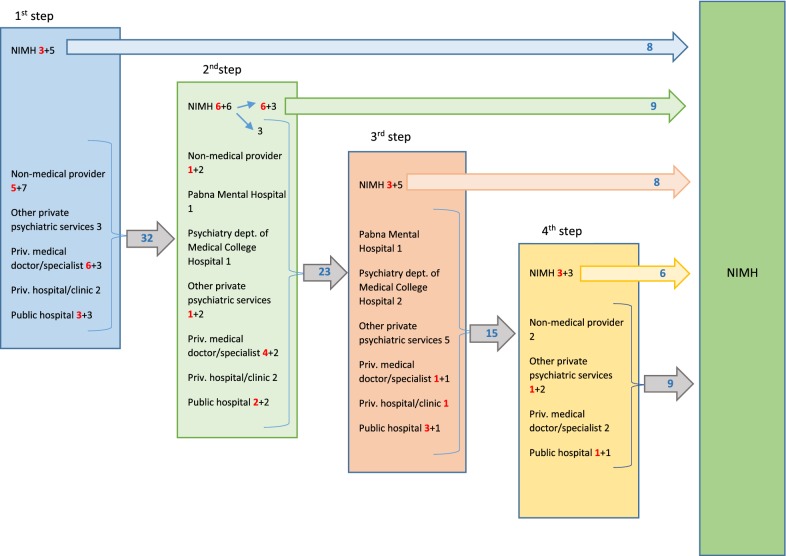
Pathways of care seeking by mental health patients visiting NIMH (numbers in red represent minor patients, in black adult patients and in blue combined)

Figure 1 shows the care-seeking pathways up to the point the patients arrived at the NIMH. Of the participants who had consulted a psychiatric care provider i.e., psychiatrist or psychologist (11/40; 27.5%), eight had come to NIMH, two saw a private psychiatrist and one had gone to a private psychologist. Among those who had consulted non-medical care providers i.e., traditional healer/religious healer/homeopath/drug seller at pharmacy or meditation center (12; 30%), eight had gone to a traditional healer, two had seen a religious healer, one had seen a drug seller at the local pharmacy, and one had gone to a homeopath. The remaining participants (17; 42.5%), had consulted non-psychiatric medical care providers (general practitioner/other medical specialists) for services as the first step. Nine had gone to the private chambers of general practitioners or other medical specialists (i.e., gynecologist, neurologist, pediatrician, or internist). Two patients had been admitted to a private detox center for drug addiction. The remaining six patients had gone to public hospitals (i.e., local primary health care centers, medical college hospitals and specialized hospitals including a pediatric hospital, and the National Institute of Neurosciences.

#### Second step

Eight patients had reached the NIMH in their first step of care seeking. Twelve of the remaining 32 patients had had a consultation at NIMH as their second care-seeking step and nine of the 12 stayed with the NIMH for care. The other three (of the 12) switched to another category of provider after their initial NIMH consultation. Of the 20 remaining patients, one had gone to Pabna Mental Hospital, one to the psychiatry department of a medical college hospital, three to private psychiatrists, 12 to non-psychiatric medical care providers or institutions, and three to non-medical care providers.

#### Third step

There were 23 patients who had a third care-seeking step and eight of them had consulted NIMH in this step. Of the remaining 15, one had gone to the Pabna Mental Hospital, two to psychiatry the department of medical college hospital, four visited private chambers of psychiatrists, one consulted a private psychologist, and seven to non-psychiatric medical care providers or institutions. No patient (of the 23) sought care from a non-medical care provider at this step.

#### Fourth and the last step

At the fourth step of the care-seeking pathway, six of the 15 remaining patients sought care at NIMH. Three of the nine other patients had received care services from a private psychiatrist, two from non-medical care providers and four from other medical care providers or institutions. The final remaining nine patients did seek care from NIMH as their fifth provider.

##### Pathways for minors

There were 17 minor respondents (< 18 years) of whom three contacted NIMH directly. NIMH was the second step in the pathway to care for six patients, the third step for three patients, the fourth step for three patients and the fifth step for two patients. A diverse range of practitioners were consulted at the interim steps including non-psychiatric medical care providers, non-medical providers and private psychiatric care providers.

##### Pathways for adults

Of the 23 adult respondents five came to NIMH directly, three in the second step, five in the third step, three in the fourth step and seven in the fifth step of the pathway to care. Most of them also consulted a diverse range of practitioners at the interim steps including non-psychiatric medical care providers, non-medical providers and other psychiatric care providers.

### Key issues emerging from pathway analysis

#### Steps taken to reach a psychiatric care provider

A total of 11/40 (27.5%) patients reached a psychiatric care provider in the first step of their care-seeking pathway, followed by 15 (37.5%) in their second step, eight (20%) in their third step, five (12.5%) in their fourth step and one (2.5%) in his/her fifth step.

Among the 17 minor patients three consulted psychiatric care providers in the first step, followed by seven in the second step, two in the third step, four in the fourth step and one in the fifth step. Of the 23 adult patients eight contacted psychiatric care providers in the first step of their care-seeking pathway, eight in the second step, six in the third step and one in the fourth step.

#### Referral pattern

Approximately one-third of the patients (13; 32.5%) had been advised to go to the NIMH by a private physician/a hospital/a psychiatrist during their care pathway. But none of those patients had received a formal referral with appropriate documents. One attendant stated the following:

*“First I took him to the nearby Upazila Health Complex (UHC). The doctors there suggested me to take him to a psychiatrist.” (Father of an 18*-*year*-*old male patient suffering from schizophrenia)*

One patient had been advised by a traditional healer to seek care from the NIMH. Thus, the majority of the patients were self-referred to the NIMH.

##### Parallel consultations from various providers

Three of the 40 participants had consulted NIMH psychiatrists in their private chambers, in addition to receiving some care from this hospital. The reason for this parallel consultation was that the patients preferred receiving better attention and longer consultations in the psychiatrist’s private chamber, but their ability to pay was weak. So the psychiatrists suggested that the patients go to the NIMH outpatient department to receive free medications and/or free psychotherapy, and be admitted to the inpatient department when their condition became acute. An attendant stated:

*“She was under his treatment for about 6* *months. Later on he said that she has to take therapy. I am not rich, so the doctor advised me to take free therapy from here. After that we have been also coming here.” (Husband of a 30*-*year*-*old female patient suffering from somatoform disorder)*

An additional three participants had received parallel consultations: two from a homeopath and one from a neurologist while also receiving care services from NIMH. They stated they lacked trust in the NIMH treatment since they had not observed expected improvement in their condition. The following quote illustrates this theme:

*“I receive mainly the therapies and sometimes other treatments from here. I also consult psychiatrists of NIMH in their private chambers and four different neurologists time to time, because the medications prescribed by the psychiatrists are not improving my condition. Actually none of the treatments is solving my problem. I don’t have peace in my mind. I want to be cured, so I go to every kind of doctor I think might be helpful.” (25*-*year*-*old male patient suffering from non*-*organic insomnia)*

##### Switching to a non-medical care provider after consulting a psychiatrist

In the care-seeking pathway, two participants had switched to a non-medical care provider after receiving care from a psychiatrist; one had gone to a traditional healer and the other to a homeopath. Both stated they had a lack of trust in the consulting psychiatrist since they had relapsed after stopping their medication. One attendant mentioned:

*“My son got really better by treatment and so stopped taking the medicaments. Even if we insisted we could not make him take those medications. This was a big mistake. As a result, his symptoms relapsed. He started behaving crazy again. Some of my neighbors in my village suggested a traditional healer. They said that modern medications cannot cure his disease and would just subside temporarily. So I took him to a traditional healer.” (Father of an 18*-*year*-*old patient suffering from schizophrenia)*

### Number of patients consulting each provider category

The majority of the patients consulted more than one care provider in a wide range of provider categories. On average, the patients consulted three different categories of provider. We calculated the total number of patients who consulted each category of provider at all steps in their care-seeking pathways (see Table 2). The largest number of patients (12) had consulted private psychiatrists, whereas one patient had consulted a psychologist for care services. For non-medical caregivers, traditional healers were the most common (nine patients) and religious healers were next (four patients). For non-psychiatric medical care providers, private neurologists were consulted by seven patients and caregivers at the National Institute of Neurosciences and Hospital by four patients.

**Table 2:** Number of patients consulting each category of provider in any step of their care-seeking pathway (multiple responses per patient possible)

Provider groups	Provider category	Number of patients choosing this consultation
Non-medical provider	Traditional healer	9
	Religious healer	4
	Drug seller at pharmacy	1
	Homeopath	2
	Meditation (quantum method)	1
Other medical provider (private)	General practitioner	4
	Neurologist	7
	Gynecologist	1
	Pediatrician	3
	Internal medicine	2
	Cardiologist	1
	Respiratory physician	1
Private clinics	Clinic	1
	Detox center	2
	Rehab	2
Public general hospitals	Upazila health complex	2
	National Institute of Neurosciences and Hospital	4
	Dhaka Shishu Hospital (children’s hospital)	4
	Other specialized hospitals	4
	Medical College Hospital (non-psychiatry department)	3
Psychiatric care provider	NIMH	40
	Pabna Mental Hospital	2
	Medical College Hosp. (psychiatry department)	3
	Psychiatrist (private)	12
	Psychologist (private)	1

### Factors influencing provider selection

The decision on the appropriate care provider in the first step of care seeking was strongly based on the distance to the provider and the person making the decision for care. Just over half of the patients (21) had consulted a provider within five km as their first step. Seventy percent of the patients (28) consulted their first provider within 20 km. A few patients (7; 17.5%) had consulted a provider located more than 100 km away. For the majority of the patients (75%) the family had decided on the first provider for care services. Four other patients chose their first provider based on neighbors’ suggestions, as indicated by one attendant:

*“Then I talked to some people in my village about his problems and they told me ‘Take him to Dhaka, to the mental health institute. There is scope for tests there. Except there his problem can’t be cured anywhere. His problem is mental.’ (Father of a 35*-*year*-*old male patient suffering from schizophrenia)*

Perceived disease etiology also influenced the choice of provider. Just under a third of the minor participants (5/17), contacted a pediatrician in their private chamber or in a hospital as their first step in care seeking. The tendency to seek the care of medical practitioners was also identified amongst adult participants. This was demonstrated in the views of the husband of a patient with behavioral disturbance, who attributed her condition to complications of her pregnancy and hence sought care from her gynecologist:

*“She had caesarian section during the delivery of our child. About two and a half to 3* *months later she started having brain problems. She used to behave crazy, used to take off her clothes, leave the house, push away the kids. I thought that her behavioral problems were related to her caesarean section, so I took her to that doctor, her gynecologist.” (Husband of a 30*-*year*-*old female patient suffering from somatoform disorder)*

For one-fourth of the total participants (10), mental health problems were initially considered to be the effect of a bad spirit. In these cases, the patients or their family preferred to contact a traditional or religious healer. In the quote below, a patient explains how others initially interpreted his problem:

*“People used to say that I was possessed by Jinni, by ghost and so on. (weeping) When I used to go to school, by riding bicycle, some villagers used to shout, “Jinni is coming.” Many people used to avoid me. At first my parents took me to a religious healer. He gave me “Tabij” and did some rituals, like he burnt the dried coconut skin and blew the smoke into my eyes. Naturally it didn’t improve my condition.” (25*-*year*-*old male patient suffering from non*-*organic insomnia)*

For nine patients, their mental health problems were perceived to be solely due to a malfunction of their brain, so they preferred consulting a neurologist. An attendant provided the following description:

*“Few months back I took him to another hospital nearby. A neighbor told me that they do brain test for free and I should let my son’s brain be checked. So I took him there. I thought that by tests they can identify the defect in his brain and can cure it. In my mind I always think how my son could be cured completely, where could I get such treatment?” (Mother of a 28*-*year*-*old patient suffering from a personality disorder)*

### Duration of delays in the care pathways

The time passed from the start of symptoms until the first contact with a provider ranged from a few hours to 15 years with a median delay of 6 months and a mean delay of 21 months. The group of patients who took a direct care-seeking pathway to a psychiatric care provider had the longest delay of 12.6 months on average. Those who consulted a non-psychiatric medical care provider at first had the shortest delay of 3.2 months on average. The patients who first consulted a non-medical care provider had a mean delay of 7.4 months. Seventeen (42.5%) patients had a delay of more than 6 months before contacting their first care provider (in any category).

Among the 32 patients who had consulted more than one provider, 14 (43.8%) had more than 6 months’ delay at any step of their care pathway when changing providers, and the maximum delay of this kind was 15 years.

The delay between the onset of symptoms and first contact with any psychiatric care provider ranged from 1 week to 15 years with a median of 1 year and a mean delay of 39.6 months. Twenty-six patients (65%) had had a delay of more than 6 months in reaching any psychiatric care provider for care.

In case of the 17 minor patients, the delay between the onset of symptoms and the first contact with a care provider ranged from a few days to 15 years with a median delay of 5 months and a mean delay of 18.6 months. The delay in reaching psychiatric care ranged from 1 month to 15 years, with a median delay of 12 months and a mean delay of 42.7 months.

In case of the 23 adult patients, the delay between the onset of symptoms and the first contact with a care provider ranged from a few hours to 14 years with a median delay of 6 months and a mean delay of 23 months. The delay in reaching psychiatric care ranged from 1 week to 14 years, with a median delay of 12 months and a mean delay of 37 months.

### Reasons for delay in seeking care

We grouped the original respondent statements by reasons for delay in seeking care. The most common reasons (1/4 respondents) were, (1) a lack of knowledge about mental health problems, (2) a lack of information about the place for appropriate care, and (3) not considering the problem to be serious enough to seek care. The lack of knowledge of psychiatric treatment was a common theme, as illustrated in the following quote by an attendant:

*“I became penniless by paying those traditional healers and religious healers, but my son didn’t improve at all. With huge frustration I came back to Dhaka with my son. I didn’t know where to go now for his treatment.” (Father of a 16*-*year*-*old male patient suffering from bipolar disorder)*

Each of the following reasons were mentioned by three respondents: (1) financial constraints, (2) no one at the household to take responsibility of care seeking, (3) thought or were told that their problem would be cured over time, (4) the patient was quite fine during the interval between treatments, and (5) the drug addicts enjoyed taking drugs and didn’t want to stop, so they were reluctant to seek treatment. The financial barriers to care was a common theme, as indicated in the following view expressed by an attendant:

*“My husband left me when I was pregnant with this child. He married again and didn’t keep any contact with us. My husband has come back to me 8* *years back, when he fell very ill and couldn’t work anymore. I had to take care of him and borrow money for his treatment. He also has that wife. I had no financial capability to treat my child.” (Mother of a 17*-*year*-*old female patient suffering from mental retardation)*

Two patients were non-cooperative and resistant to any attempt to seek care from a doctor, and one became abusive and violent when forced. An attendant mentioned the following experience:

*“I brought her to Dhaka and took her to neuroscience hospital. But there I could not take her into doctor’s room. I bought outpatient ticket and waited for some hours but could not convince her. We also tried to take her to the doctor’s room by force but she turned violent. She started scolding with slang words and was hitting us. Then she left the hospital by force. So we could not get her any consultation there.” (Brother of a 45*-*year*-*old female suffering from schizophrenia)*

Two patients had difficulty securing public transport to the care provider’s distant location, which resulted in a delay. The attendant of one of those two shared about the following challenges:

*“I have to take leave from my office and go to our village to pick her up and to bring her back. She cannot travel with anyone else, particularly with a male, because she has to be accompanied to washroom on the way. It’s a long trip. In the washrooms of highway restaurants, it is not allowed that a male would enter female washroom. Often I cannot find a known female who could travel with her.” (Daughter of a 65*-*year*-*old female patient suffering from dementia)*

Attendants of two other minors said that the head of household was negligent about the child’s treatment and made no effort to seek care. One patient mentioned that he was too young to understand his own mental health problem when it began during childhood, so he didn’t speak about it with his family, neither he himself could have sought care at that time. Another patient lacked the time for follow-up visits due to his work schedule. Two patients mentioned feeling hopeless due to a lack of improvement after previous treatments and so had lack of continuity of care. This quote from an attendant expressed her lack of hope:

*“I took him there for about three months and didn’t continue because I didn’t see any improvement of my son’s condition. I got frustrated and used to feel very sad about his problem.” (Mother of an 11*-*year*-*old male patient suffering from autism)*

One respondent was confused by the various diagnoses and treatments provided by several providers, which resulted in a delay in her further treatment. Another patient believed she couldn’t rely on her provider, and so discontinued her treatment. The father of one patient stated that the reason for the delay in his seeking care was that his son had a final exam at school and he didn’t want to disturb his studies by taking him to a far place to consult a psychiatrist. Two patients were told by their provider that they had no mental health problems, so they did not require treatment.

Two patients had no delay or interruption in care seeking, although they finally reached a psychiatric care provider after delays of 2 years and 5 months, respectively. Both of these patients consulted four categories of providers before coming to NIMH for care, and both sought information on the right provider since the onset of their symptoms to find relief. The following quote is from the attendant of one of these two:

*“I got her admitted to the hospital again. At that time, I told the doctors, “My patient’s condition is not improving. So please refer her to somewhere else, to Dhaka. Why this is happening? You have done all kinds of tests; MRI, digital X*-*ray, CT scan. No abnormality could be found. All reports were normal. So please give me a good advice about what to do.” Doctors said, “No, you don’t need to go somewhere else. Let her treatment be continued here and she will get better.” I said, “No sir. Please refer my patient to Dhaka. You know where it will be good to take her. Please refer her there.” At that time the doctors didn’t agree and didn’t refer her.” (Mother of a 16*-*year*-*old female patient suffering from dissociative disorder)*

## Discussion

We chose a mixed-method design for this study to generate a wider view and understanding of the mental health care-seeking pathways and the various factors influencing these pathways for patients receiving care at the NIMH. To our knowledge, only one study has been previously conducted about pathway to psychiatric care in Bangladesh and that one used a quantitative approach [16]. Our main study findings are as follows.

1. As the first care provider, non-psychiatric medical care providers were predominant, and non-medical providers were second most frequently consulted.
2. Private psychiatrists were the most visited individual category of care providers, followed by traditional healers.
3. Institutional referral rates to psychiatric care providers by non-psychiatric medical care providers were low and informal (no referral documents provided for any participant).
4. There is a lack of mental health services available at the community/primary level of care.
5. Family choice, distance to the provider and the perceived etiology of the disease were the main reasons for choosing a specific provider.
6. The mean delay in care seeking was long (from the onset of symptoms until the first contact with any provider 21 months and until the first contact with a psychiatric care provider 39.6 months), and the most common reasons for delays were the lack of information and education about mental health.

A few studies on pathways of psychiatric care have been conducted in Asian tertiary hospitals including Bangladesh, India, Nepal, Japan and Mongolia [16, 17]. A comparison of the main findings of these studies and our study is presented in Table 3.

**Table 3:** Category of first consulted health provider by patients with mental health problems in various Asian countries as reported in two studies and compared to this study’s findings

Category of provider first consulted	Our 2016 Bangladeshi study	2008 Bangladeshi study [16]	India [17]	Nepal [17]	Japan [17]	Mongolia [17]
	% of participant provider choices					
Psychiatric care provider	27.5	16.0	42.0	2.0	32.0	54.0
Non-psychiatric medical care provider	42.5	44.0	28.0	38.0	64.0	16.0
Traditional/religious healer	25.0	22.0	20.0	14.0	–	8.0

In our study, 5% of the patients reported receiving treatment from a homeopath. In a 2015 study in Nepal, 8% of the patients had consulted alternative medicine providers (homeopathy, naturopathy, Unani, etc.) [17]. In contrast, in a previous Bangladeshi study in 2008 [16], no patient had consulted an alternative medicine provider, although 12% of the patients in this study had received care from rural medical practitioners (non-qualified medicine practitioners) [16]. Rural medical practitioners were not mentioned by any respondent in our study.

The Bangladeshi study had been conducted in 2008 and recruited 50 newly registered subjects with new episodes of psychiatric illness [16], irrespective of their distribution across disease categories in the outpatient psychiatric department of a medical college hospital [16]. Our study conducted in 2016 in the Bangladesh national specialized mental hospital, selected four patients in each of the ten ICD 10 categories for mental health, thus, the findings of the two care pathway studies are not fully comparable due to methodological differences.

In our study, the number of patients first consulting non-medical care providers was higher than those who had consulted a psychiatric care provider. In Asian countries, traditional and religious healers play a vital role in the mental health care pathway [15]. According to a national mental health survey in India [22], most patients with mental illness had undergone unnecessary treatment, mainly faith healing, before receiving professional care. The general tendency in India was to consult a local priest first and then, if no improvement, to visit a local doctor and later a psychiatrist [22]. In a WHO report on Bangladesh in 2015, the main factor influencing the choice of an informal health care provider in general over a formal provider was easy access, low-cost of treatment and availability of the provider on demand [11]. This provider is often a member of the community with informal relations with the patients or is a family relative. Therefore, people may trust them more and feel comfortable consulting them for private matters.

In our study, parallel consultations and switching to non-medical provider from a psychiatric care provider resulted from dissatisfaction regarding the quality of consultation provided at the NIMH outpatient unit and treatment outcomes. Negative perceptions of the quality of health care in Bangladesh make people reluctant to seek care [11]. Moreover, many mental illness are chronic, such as schizophrenia, mental retardation, personality disorder, and require long-term and sometimes life-long care. Some mental illness have recurrent acute episodes, such as mood disorders, anxiety disorders [23]. Therefore, some patients may lose trust in stand-alone psychiatric treatment and choose other providers for simultaneous care. Parallel care seeking was also found in 29% of the patients in the 2008 Bangladeshi study [16].

In Bangladesh, there is no structured health care referral system [14], so patients choose a convenient provider according to their availability, accessibility and affordability [11]. Mental health patients have direct access to psychiatric services in Bangladesh, unlike most Western European countries; hence general practitioners and hospitals are not gatekeepers [17]. In India, primary health care professionals are often inadequately trained, and reluctant or unable to detect, diagnose or manage common mental disorders according to a national survey conducted in 2015–2016 [22] and we believe the same is true in Bangladesh [14]. Considering our finding that only one-third of the patients had been suggested to come to the NIMH by a general practitioner/hospital/psychiatrist, action is needed to establish a well-functioning referral system for mental health patients in Bangladesh.

People in Bangladesh generally consult a provider within a convenient distance. Transportation of a patient with mental illness, particularly in acute condition, is associated with special challenges. Almost no mental health care services are available at a primary or secondary level of care in Bangladesh [14]. Since unskilled care providers are more dominant in the rural areas of Bangladesh compared to urban areas [11], for many rural communities there is no choice for mental health care other than non-medical providers. The 2015 WHO report and a 2005 Bangladeshi study on general health care services reported a high percentage of patients receiving health services from an informal provider, 60% [11] and 52% respectively [24]. To ensure access to proper mental health care for the majority of the rural population in Bangladesh, an integration of mental health care at a primary level of the health system is most urgent.

Family plays a vital role when it comes to the choice of provider for Bangladeshi patients with mental illness in this study. These patients are often unable to make decisions regarding their care services during acute episodes, so, their families decide whom to consult. Similar findings were reported in the 2008 Bangladeshi study [16]. In addition, the choice of a particular category of provider is also influenced by beliefs about the disease etiology and expectations regarding the efficacy of a particular type of therapy [16]. More than half of the respondents in a 2006 nationwide survey in Bangladesh believed myths about the supernatural causation of mental illness and almost half of the respondents said that they would prefer traditional treatment and religious rituals for treating mental illness [7].

Compared to our study findings on delays seeking care in general at the onset of mental health symptoms (29 weeks), the mean delay in the 2008 Bangladeshi study [16] was lower, (14 weeks). In India, this particular delay ranged from 2.5 months to 1 year depending on the type of mental illness [22]. Our study had a mean delay in reaching a mental health professional of 75 weeks, which was higher than the finding of the 2008 Bangladeshi study, which had a mean of 48 weeks. Almost half of our study patients had consulted a non-psychiatric medical care provider at the first onset of symptoms, and this group had the shortest delay (14 weeks). In contrast, the 2008 Bangladeshi study found that patients consulting a non-psychiatric medical care provider first had the longest delay of all patients from the onset of symptoms (22–31 weeks). So timely and active referral by non-psychiatric medical care providers would reduce patient delays in reaching psychiatric care providers in Bangladesh.

In Bangladeshi society, mental health problems are associated with strong stigma and lack of awareness [11, 14, 25], which are the barriers for access to care services [7, 22]. A study conducted in the capital city of Bangladesh [26], in 2008, concluded that a lack of knowledge about mental illness symptoms was responsible for more than two-thirds of the delays (69%). Other reasons included social stigma (12%), delays by doctors (8%), belief systems (8%) and the lack of social and financial support (3%). In a study in India, only 10% of the mental health patients received evidence-based treatment due to obstacles related to stigma [3]. Erroneous perceptions about mental illness in the Indian community resulted in overall delays in help seeking and the choice of a non-medical care provider [22]. Therefore, even if mental health care is made available at all levels in Bangladesh, it is possible that persistent stigma and discrimination will continue to hamper access to psychiatric care. It is critically important to build mental health literacy and implement strategies to combat the lack of knowledge, stigma and discrimination for the whole Bangladeshi population [5].

### Study strengths and limitations

As the study was conducted at a tertiary referral center, there was an inevitable selection bias that influenced the types of respondents engaged in the study. The pathways to psychiatric care for patients who do not require referral to NIMH were not explored. Furthermore, recall bias may have affected the results as the study design required patients and their attendants to provide a retrospective view of their pathways to care. A larger population based study would be worthwhile to assess pathways to mental health care more broadly. On the other hand, there is lack of research about mental health systems in Bangladesh and our study findings provide valuable information about this area. A mixed method design has facilitated the generation of more detailed information about various aspects of the care pathways.

## Conclusions

This study explored various aspects of care-seeking pathways of patients with mental health problems and influencing factors. We conclude that the care pathways of these patients in Bangladesh are often unnecessarily long due to consultations with a variety of provider categories before reaching a psychiatric care provider. There is lack of information and education in the general population regarding mental health and appropriate care. So, non-medical providers play an important role in the care pathway. Moreover, there is a lack of institutional referrals and mental health care availability at the community/primary levels. All these factors result in delays in mental health care.

It is true that the mental health care situation is poor in Bangladesh. But in recent years there have been some progress. World Mental Health Day is observed through elaborate programs by various social, psychiatric and psychological associations, and print and electronic media every year with a highlighted theme [27] to raise public awareness. Training courses are being undertaken to educate traditional and religious healers in Bangladesh about mental health [17]. Improving the knowledge of those providers would hopefully enable them to understand their limitations in treating mental illness and encourage them to refer patients to appropriate mental health care providers. Patient referrals by traditional and religious healers to psychiatric services has been proven to be successful in some regions of Bangladesh [16]. Resources for mental health institutions like NIMH are also being increased from time to time to enable additional and improved service provision.

A blueprint for community-based mental health services has been jointly developed by Bangladesh government and WHO. NIMH then provided a justification and description of the advantages and feasible ways of providing mental health services through primary health care as a viable method to close the treatment gap. The main themes of this strategy were to develop trained non-specialist workers and referral and back-referral programs with occasional shared care approaches in model *upazilas* (sub-districts) in the context of a limited number of specialist mental health service providers and the absence of separate community mental health centers [11]. An urgent implementation of this plan would improve access to proper mental health care and would reduce delays in care pathways. Western specialists have suggested a similar approach to reduce mental health treatment gaps [5]. Those specialists have also suggested the inclusion of integrated care for mental illness along with other non-communicable diseases [3].

Considering the mental health care needs in Bangladesh, current efforts are insufficient and progress is not adequate. Improving the availability of mental health care at community/primary levels and institutional referrals, and increasing the number of psychiatric care providers at higher levels of health care are urgently needed. Recently, mental health was included in the UN Sustainable Development Goals, and for the first time in history, it has been recognized as a health priority within the global development agenda [28]. This recognition is believed to have had a positive influence on mental health care systems development in member countries [29]. By being part of the global development agenda, mental health is expected to receive more attention and thus more resources and increased monitoring, particularly in low-income countries like Bangladesh. It is hoped that eventually, these strategies will improve mental health care access for all those in need across the country.

## Abbreviations

NIMH: National Institute of Mental Health
WHO: World Health Organization
OPD: outpatient department
EU: European Union
ICD: International Classification of Diseases
UN: United Nations
